# Impacts of genomic research on soybean improvement in East Asia

**DOI:** 10.1007/s00122-019-03462-6

**Published:** 2019-10-23

**Authors:** Man-Wah Li, Zhili Wang, Bingjun Jiang, Akito Kaga, Fuk-Ling Wong, Guohong Zhang, Tianfu Han, Gyuhwa Chung, Henry Nguyen, Hon-Ming Lam

**Affiliations:** 1 Centre for Soybean Research of the State Key Laboratory of Agrobiotechnology and School of Life Sciences, The Chinese University of Hong Kong, Shatin, Hong Kong Special Administrative Region China; 2 Ministry of Agriculture Key Laboratory of Soybean Biology (Beijing), Institute of Crop Sciences, The Chinese Academy of Agricultural Sciences, 12 Zhongguancun South Street, Beijing, 100081 China; 3 Soybean and Field Crop Applied Genomics Research Unit, Institute of Crop Science, National Agriculture and Food Research Organization, Kannondai 2-1-2, Tsukuba, Ibaraki 305-8518 Japan; 4 Institute of Dryland Agriculture, Gansu Academy of Agricultural Sciences, Key Laboratory of Northwest Drought Crop Cultivation of Chinese Ministry of Agriculture, Lanzhou, 730070 China; 5 Department of Biotechnology, Chonnam National University, Yeosu, Chonnam 59626 Korea; 6 Division of Plant Sciences and National Center for Soybean Biotechnology, University of Missouri, Columbia, MO USA

## Abstract

It has been commonly accepted that soybean domestication originated in East Asia. Although East Asia has the historical merit in soybean production, the USA has become the top soybean producer in the world since 1950s. Following that, Brazil and Argentina have been the major soybean producers since 1970s and 1990s, respectively. China has once been the exporter of soybean to Japan before 1990s, yet she became a net soybean importer as Japan and the Republic of Korea do. Furthermore, the soybean yield per unit area in East Asia has stagnated during the past decade. To improve soybean production and enhance food security in these East Asian countries, much investment has been made, especially in the breeding of better performing soybean germplasms. As a result, China, Japan, and the Republic of Korea have become three important centers for soybean genomic research. With new technologies, the rate and precision of the identification of important genomic loci associated with desired traits from germplasm collections or mutants have increased significantly. Genome editing on soybean is also becoming more established. The year 2019 marked a new era for crop genome editing in the commercialization of the first genome-edited plant product, which is a high-oleic-acid soybean oil. In this review, we have summarized the latest developments in soybean breeding technologies and the remarkable progress in soybean breeding-related research in China, Japan, and the Republic of Korea.

## Introduction

Cultivated soybean (*Glycine max* [L.] Merr.) is believed to be domesticated from annual wild soybean (*Glycine soja*) thousands of years ago (Carter et al. 2004; Hymowitz 1970). The question of whether cultivated soybean originated from a single (Jeong et al. 2019; Zhou et al. 2015) or multiple domestication events (Lee et al. 2011a; Xu et al. 2002) remains unanswered. However, it is generally agreed that ancient China, Korea, and Japan were the three pioneering societies to domesticate and cultivate soybean (Hymowitz 1970; Jeong et al. 2019; Lee et al. 2011a; Sedivy et al. 2017; Xu et al. 2002).

**Fig. 1:**
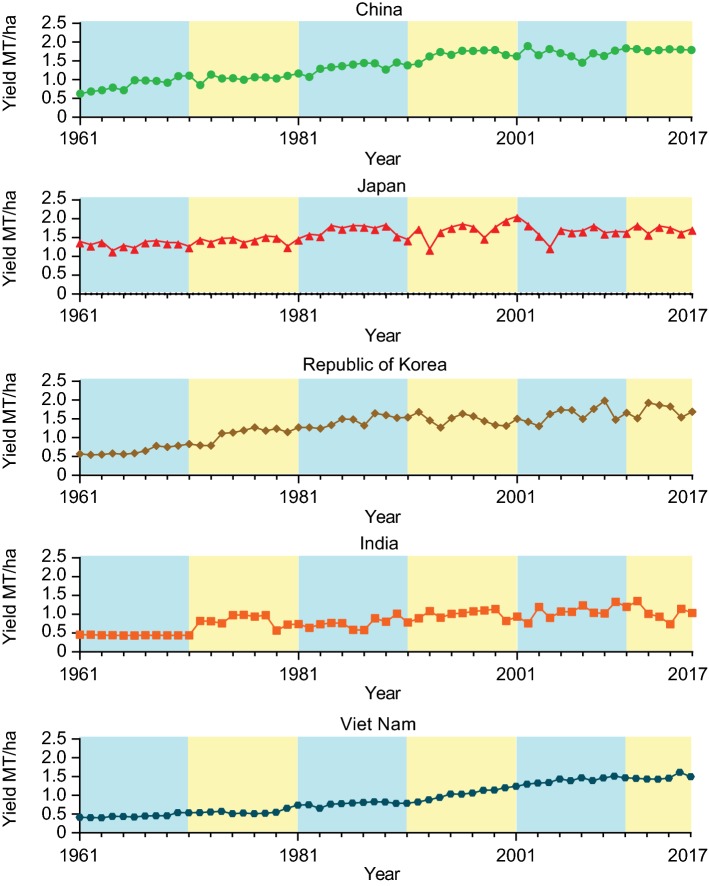
Per-hectare yield of soybean in selected Asian countries. Data were obtained from FAOstat (retrieved on May 20, 2019, from http://www.fao.org/faostat/en/#home)

As a major economic crop of high plant oil and protein contents, there has been an escalating demand for soybean. Consequently, increasing soybean yield has always been the main objective of crop improvement. With the advents of modern farming practices and genetic improvements, the yield of soybean has steadily increased in Asia since 1961 (Fig. 1). The soybean yield in China increased from 0.6 metric tons per hectare (MT/ha) in 1961 to a record high of 1.89 MT/ha in 2002 and then plateaued at around 1.8 MT/ha for 15 years after (Fig. 1). Similarly, the soybean yield in Korea steadily increased from 0.6 MT/ha in 1961 to a peak of 1.98 MT/ha in 2009 and has stabilized at an average of 1.7 MT/ha in subsequent years (Fig. 1). The yield of soybean in Japan was 1.3 MT/ha in 1961, doubling those of the nearby countries during the same period. Nevertheless, the increase in soybean yield in Japan has occurred at a slower pace and the yield is now at a similar level to those in China and Korea (Fig. 1). Seemingly, the soybean yield in China, Japan, and Korea and nearby countries such as Vietnam and India has reached a plateau in recent years as well (Fig. 1). The stagnation of soybean yield growth in these countries could be due to multiple factors such as the relocation of soybean production from optimum (like Middle Northeast China) to harsh environments (like high-latitude cold regions in China) and the limitations in soil fertility and water resources. To further boost the soybean yield, improved soybean cultivars with higher yield potential and stronger tolerance to environmental stresses may provide a partial solution. However, the genetic bottleneck has hampered the progress in soybean breeding over the last century (Hyten et al. 2006). The recent rapid development in genome sequencing technology, the release of high-quality genome sequences, and a better understanding of the genomic regions associated with agronomically important traits provide important tools for accelerating the progress of marker-assisted and molecular breeding programs in soybean.

## Milestones of soybean genomic research in the three original soybean-domesticating countries

The first reference genome of the cultivated soybean is based on the cultivar, Williams 82 (Wm82) (Schmutz et al. 2010), which has been extensively used in various soybean genomic studies in the past decade (Chung et al. 2014; Kim et al. 2010; Lam et al. 2010; Li et al. 2014; Qi et al. 2014; Qiu et al. 2014; Zhou et al. 2015). However, Wm82 is a cultivated soybean bred in North America (Schmutz et al. 2010) and is distinct from those in South America and Asia (Vaughn and Li 2016; Wolfgang and An 2017; Zhou et al. 2015). Therefore, using Wm82 as the only reference for population genomic analyses may not be sufficient to capture all the genetic variations from diverse origins. As a result, genome assemblies of soybean accessions from different regional niches or ones bred for unique purposes were built.

In order to characterize the genomic structure of Japanese soybean, the genome of a leading Japanese cultivar Enrei was sequenced (Shimomura et al. 2015). Enrei is a breeding variety released in 1971 with the aim to boost the soybean production in central Japan (Shurtleff and Aoyagi 2014). It quickly became a leading cultivar with a high seed quality for food processing (Shurtleff and Aoyagi 2014). Enrei is therefore the major soybean used in soybean research in Japan. Approximately two million single-nucleotide polymorphisms (SNPs) and insertions/deletions (INDELs) were identified between Enrei and Wm82 (Shimomura et al. 2015). The sequence information of Enrei is available at DAIZUbase (Katayose et al. 2011). Information from the Enrei genome has been used for gene discovery and, later, for breeding purposes. A chromosomal segment substitution lines (CSSLs) collection was produced by crossing Enrei with the Chinese landrace, Peking (Watanabe et al. 2018). Peking is a founder line for many disease-resistant cultivars. The low-complexity CSSL collection, with Peking genome segments in the Enrei background, could facilitate the identification of useful genetic resources from Peking and reduce the undesired attributes of the Peking background in later breeding manipulations. Furthermore, an ethyl methanesulfonate (EMS) mutant library of Enrei has been made (Tsuda et al. 2015). After two rounds of mutagenesis, 1437 M2 plants were able to set viable seeds with an average mutation density of 1 mutation/74 kb (Tsuda et al. 2015). This mutation rate should be enough for identifying mutants for forward genetic studies. Other than Enrei-based researches, the resequencing of Japanese and world soybean mini-core collections (Kaga et al. 2011) is also ongoing in Japan. Meanwhile, resequencing using AmpliSeq technology has identified novel variants of flowering time-related genes from the Japanese mini-core consisting of 96 soybean accessions, of which 65 are of Japanese origin (Ogiso-Tanaka et al. 2019).

Korea was among the pioneers to carry out deep sequencing of undomesticated soybean accessions using next-generation sequencing (Kim et al. 2010). A Korean wild soybean IT182932 was sequenced at a high depth of ~43-fold sequence coverage. By mapping the resequencing reads to the Wm82 reference genome and assembling the unmapped reads *de novo*, it was found that the genomes of the wild soybean and the cultivated one differ by around 3.76% (Kim et al. 2010). The wild-specific contigs of IT182932 were used in subsequent population genomic analyses in the absence of a wild soybean reference genome (Lee et al. 2013; Zhou et al. 2015). The INDEL information from IT182932 deep sequencing has been used to build a high-density linkage map of an F15 population derived from *G. max* Hwangkeum and *G. soja* IT182932 (Lee et al. 2013), providing supporting evidence that introgression is not uncommon in soybean. The resequencing approach enabled the marker interval to be reduced to under 6 cM, and consequently, these authors were able to identify seven previously misplaced markers, two absent ones, and six INDEL markers placed on the scaffolds in the Wm82 assembly owing to the increased resolution (Lee et al. 2013). Later, a resequencing study on nine Korean *G. max* accessions and five Korean *G. soja* accessions was also able to assemble unmapped reads to contigs for gene identification (Chung et al. 2014). In total, 1326 putative genes were identified in 31 Mb of *de novo* contigs and at least 343 of them showed a high degree of homology with known plant proteins (Chung et al. 2014), providing further support that some genes are conserved in wild germplasms.

A few hallmark resequencing studies have been done in China (Lam et al. 2010; Li et al. 2013; Zhou et al. 2015). Efforts have also been made to assemble draft genomes of wild soybeans and semi-wild soybeans (Qi et al. 2014; Qiu et al. 2014). Although the causal gene for salt tolerance was obtained through one of these draft genomes (Qi et al. 2014), the contiguity of these genomes makes them ineffective for genome comparisons. In a hope to better represent the diversity of wild soybeans, a pan-genome of seven geographically distinct *G. soja* accessions was also built (Li et al. 2014). The pan-genome has a size of 986.3 Mb, consisting of 59,080 annotated gene families. More than 20% of the pan-genome is dispensable, presumably related to environmental adaptations. The dispensable portion of the genome consists of a collection of genes for crop improvement. Yet with all this new information, the pan-genome is still far from being usable in breeding applications.

Recently, there are two breakthroughs in soybean genomic research in China: the assembly of a high-quality reference genome of Zhonghuang 13 (ZH13), a popular Chinese cultivated soybean and that of a high-quality reference genome of a wild soybean W05 (Shen et al. 2018; Xie et al. 2019). ZH13 is a bred released in Anhui Province, China, in 2001 as a multi-resistant soybean cultivar and became the most popular soybean cultivar in China since 2007 (Chinese Academy of Agricultural Sciences 2016). The cumulative cultivated area under ZH13 has reached 6.7 million hectares in 2018, demonstrating its importance in Chinese agriculture (Wei 2018). By using a bundle of state-of-the-art technologies, including PacBio single-molecule real-time sequencing, Hi-C sequencing, and optical mapping, the genome of ZH13 was assembled into 21 superscaffolds corresponding to the 20 chromosomal and the chloroplast genomes (Shen et al. 2018). The total assembled size of the genome is 1025 Mb with the contig N50 of 3.46 Mb. Assembled genome of ZH13 shows 84% synteny with Wm82. There are 1404 translocation events and 12,170 present–absent variations (PAVs) between the American and Chinese soybean reference genomes. The discrepancies may be due to the advancement in technologies between the two generations of genome assembly. Regardless, the variations between Wm82 and ZH13 also show that a single reference genome is not sufficient to capture all the genomic variations across soybeans of different geographical origins.

The other breakthrough in soybean genomic research is the release of the first reference-grade genome of the wild soybean, W05 (Xie et al. 2019). Wild soybean has been shown to be the reservoir for rich genetic resources (Chung et al. 2014; Lam et al. 2010; Li et al. 2013, 2014; Qiu et al. 2014; Zhou et al. 2015). Since the release of the Wm82 genome, there have been a few attempts to assemble the genomes of wild soybeans (Li et al. 2014; Qi et al. 2014; Qiu et al. 2014). Yet these assemblies are too fragmented for effective analyses. The genome of W05 was the first complete reference genome for wild soybean. W05 exhibits typical wild soybean morphologies (Lam et al. 2010; Qi et al. 2014). Thus, the reference genome of W05 could possibly be used to study the genomic differences between wild and cultivated soybeans. In fact, the genomic variation that caused the transition in seed coat color as a result of domestication has been identified using the W05 genome (Xie et al. 2019).

The existing genome assemblies represent soybeans from different geographical regions and backgrounds. In summary, Wm82 was released in North America in 1981 (Registration of Crop Cultivars 1988). It is a relatively old variety and has been used as the immediate parent for over 100 newer cultivars in the Americas (www.soybase.org). Thus, it is a good reference for marker identification and genetic mapping involving accessions with related pedigrees. However, breeding and research are catching up in East Asian countries in the past decade. Although in comparison with Wm82, the genome assembly of Enrei is not of reference grade, it is nevertheless a historical Japanese cultivar bred specifically for local conditions and therefore useful as a reference genome for further soybean research in that country. On the other hand, ZH13 is a widely planted cultivar in China after the year 2000, and the release of the ZH13 genome has provided a very important genetic resource for breeding high-quality soybean cultivars. W05 was an undomesticated soybean. Although the diversity of wild soybean is much higher than that of cultivated soybean, W05 possesses many typical wild traits and therefore can be used as a representative wild soybean genome to study the processes of domestication and to identify useful alleles from wild accessions.

## Recent development in soybean breeding technologies

For crop breeding in general, there are multiple ways of using genetic resources to achieve improvement. So far, crossing cultivars with known desired traits is the most common practice as it is relatively easy to obtain lines with desirable traits while maintaining crop productivity. However, if cultivars show low genetic diversity, their existing genes/alleles may not be able to cope with new environmental stresses. New genes/alleles may then be identified by screening the wild relatives or created by random mutagenesis. Yet, introducing a large portion of the genome of wild relatives into cultivars or generating random mutants could be detrimental to crop production, and therefore, a long breeding cycle is needed to purify the genetic background while maintaining the introduced traits. Marker-assisted breeding (MAB) for plant improvement can help speed up the process. The universal application of MAB is marker-assisted selection (MAS) and genomic selection (GS) and the principle of MAS and GS has been well documented (Collard et al. 2005; Crossa et al. 2017). As modern breeding approaches, MAS exploits the markers associated with simple traits, while GS using genome-wide markers has the potential to address complex traits and domestication (Akond et al. 2018; Zhang et al. 2018). Transgenesis is normally a quick way to introduce a new trait into the crop, but controversies surrounding this method hamper public acceptance, especially in East Asia and Europe. Currently, genome editing has been a quick, precise, and promising way for crop improvement with better consumer acceptance. With high-quality genome information, the precision in genome editing is also improved, and off-target editing can also be predicted and avoided. Nonetheless, even with new technologies, soybean breeding is still full of challenges.

The growth period of soybean is significantly governed by more than ten quantitative trait loci (QTLs). With different combination of alleles in these loci, photosensitivity, flowering time, maturation time, and flower number could vary a lot. Furthermore, the relatively long growing period of soybean allows for only 1–3 breeding cycles per year. Thus, crossing of distinct varieties would require careful planning to match the flowering time of the two parental lines and to maximize the number of generations per year. To solve these problems, a soybean breeding protocol using CO_2_-supplemented growth chambers has greatly shortened the time of each breeding cycle from over 100 days to around 70 days (Nagatoshi and Fujita 2019). The protocol shortens the flowering time by controlling the light/dark cycle and temperature and improves flower number and quality to increase crossing efficiency by supplementing the plant with CO_2_ and by reducing the seed-to-seed period by growing immature seeds (Nagatoshi and Fujita 2019).

Genome editing has been a better received molecular breeding method than transgenesis by society in general. The first soybean product produced by genome editing was announced in early 2019 and will be available on the market soon. By using the transcription activator-like effector nuclease (TALEN) technique, a new high-oleic-acid soybean cultivar has been made without introducing foreign DNA into the soybean genome (Calyxt Inc. 2019; Splitter 2019). Vegetable oil produced with this new soybean cultivar contains 80% oleic acid and has 20% less saturated fat, which makes the oil healthier for human consumption, and the shelf life of the oil is also extended (Calyxt Inc. 2019). It is expected that the high-oleic-acid soybean oil will be the first gene-edited plant product to be commercialized (Calyxt Inc. 2019; Splitter 2019).

Gene editing in somatic cells or in the hairy roots system has received a lot of attention in soybean research (Cai et al. 2015; Du et al. 2016; Jacobs et al. 2015; Michno et al. 2015; Sun et al. 2015). A DNA-free genome editing protocol using Cfp1 (Cas12a) has been developed for soybean, but it is limited to protoplasts (Kim et al. 2017). A protocol for whole plant transformation has been well documented (Liu et al. 2019). Successful stable lines have been generated to disable the *GmFT2a* family, the *GmSPL9* family, and two copies of *PEAPOD* genes to alter flowering time, root nodule number, and seed pod morphology, respectively (Bao et al. 2019; Cai et al. 2018a, 2019; Kanazashi et al. 2018). Nonetheless, the generation of stably edited lines largely relies on the soybean transformation efficiency, editing efficiency, and inheritability. So far, much effort has been made to screen the soybean endogenous U6 promoters to improve the expression of single-guide RNAs (sgRNAs) (Sun et al. 2015) and to optimize the codon usage of the cas9 gene in soybean to improve the translation efficiency of the enzyme (Michno et al. 2015). Constructs harboring multiple sgRNAs for editing multiple targets in the soybean genome are also available (Liu et al. 2019). There is a successful case of creating large deletions (1–4.5 kb) in the soybean genome using multiple sgRNAs targeting both *GmFT2a* and *GmFT5a*, genes that regulate flowering time and maturity (Cai et al. 2018b).

In addition, strategies used in other plants may also be adapted for soybean study. Transformation through pollen magnetofection (Zhao et al. 2017), biolistic bombardment (Rech et al. 2008), or the haploid induction system (Kelliher et al. 2019) can possibly help deliver the gene editing construct into germline cells and requires no explant regeneration. Furthermore, efforts have also been made to improve Cas9 activity. The original Cas9 gene was isolated from *Streptococcus pyogenes*. Research has demonstrated that heat shocking the Arabidopsis plant at 37 °C at the reproductive stage could increase the editing efficiency in pollen from 4.3–87% to over 95%, probably owing to the activation of the Cas9 enzyme at its optimal temperature (LeBlanc et al. 2018). Genome editing through the double-strand breakage of DNA by CRISPR/Cas9 is somewhat uncontrolled, which results in many alleles after editing. To correct this problem, base editing is achieved by fusing a base editing enzyme to Cas9 with no nuclease activity (Kang et al. 2018). Currently, base editing has been carried out successfully in rice and wheat (Li et al. 2018a; Yan et al. 2018).

## Germplasm collections in China, Japan, and the Republic of Korea

### Germplasm collections in China

The National Genebank (China) was established in 1986 to conserve the genetic resources of crop plants in China. It has preserved the genetic resources of 400,000 accessions of more than 200 crop species. There are currently over 40,000 soybean accessions in the Genebank, making up a significant portion of its collection (Table 1).

**Table 1:** Resource centers for soybean genetic resources available in specific countries/regions

Resource center	Country/region	Number of accessions		Website/contact method
		Description	Number	
National Genebank	China	*Glycine max*	31,432	Chinese Crop Germplasm Information System http://www.cgris.net
		*Glycine soja*	9684	
NARO Genebank	Japan/Ibaraki Prefecture	*Glycine soja*	2127	http://www.gene.affrc.go.jp/databases-plant_search_en.php
		*Glycine max*	7683	
		G. *tabacina*	8	
		G. *tomentella*	1	
		G. *wightii*	2	
LegumeBase	Japan/Miyazaki Prefecture	*Glycine soja*	715	https://www.legumebase.brc.miyazaki-u.ac.jp/top.jsp
		*Glycine max*	265	
		RILs	263	
		Mutant	28	
National Agrobiodiversity Center	Republic of Korea	*Glycine max*	19,338	http://www.genebank.go.kr www.naas.go.kr
		*Glycine soja*	3229	
Chung’s Wild Legume Germplasm Collection	Republic of Korea	*Glycine soja*	> 3000	Email: chung@chonnam.ac.kr

### Germplasm collections in Japan

Approximately 13,400 soybean accessions (accessed on May 22, 2019) including local landraces, improved cultivars, and breeding lines developed by regional Japanese agricultural research institutes or introduced from agricultural research institutes overseas are maintained at the NARO Genebank (Table 1), operated by the Ministry of Agriculture, Forestry, and Fisheries (MAFF) of Japan.

LegumeBase, maintained by the National BioResource Project (NBRP), distributes approximately one thousand wild and cultivated soybean accessions via an online system. NBRP is directed by the Ministry of Education, Culture, Sports, Science, and Technology of Japan. This project also catalogs and distributes other experimental genetic resources such as EMS mutant lines and recombinant inbred lines (RILs) with the associated genotype information.

### Germplasm collections in the Republic of Korea

The National Agrobiodiversity Center of Korea had its beginnings in 1987 when a seed management office of Rural Development Administration (RDA) was established (Table 1). Four years later, in 1991, the seed management office was upgraded to the Genetic Resource Department of the National Institute. In 2008, the Genetic Resource Department was transformed into the National Agrobiodiversity Center under the National Academy of Agricultural Sciences, part of the RDA of the Republic of Korea. In addition to the National Agrobiodiversity Center, a well-maintained and highly diverse legume germplasm collection is being held at Chonnam National University under the Chung’s Wild Legume Germplasm Collection (Table 1).

## Molecular breeding of soybean in East Asia to meet region-specific demands

### Progress on soybean molecular breeding in China

Like many other regions under soybean cultivation, the Chinese soybean yields are largely hampered by the soilborne pathogen *Phytophthora sojae*. Multiple races of *P. sojae* have been identified in China since 1989. At the same time, certain single dominant resistance genes (*Rps*) have been found to confer complete immunity toward certain races of *P. sojae*. So far though, there is no one *Rps* gene that confers absolute immunity against all *P. sojae* races, and thus, finding a broad-spectrum *Rps* gene/allele has become the Holy Grail for soybean breeding. A Chinese cultivar, Zaoshu 18, is able to resist ten out of 14 tested *P. sojae* isolates (Zhong et al. 2018b). A *P. sojae* resistance locus *RpsZS18* was mapped to a 71.3 kb region on chromosome 2, which can be traced with two newly developed SSR markers (Table 2) (Zhong et al. 2018b). Another Chinese cultivar, Huachun 18, confers resistance toward at least 12 *P. sojae* isolates (Zhong et al. 2018a). After QTL mapping and fine mapping, a *P. sojae* resistance locus *RpsHC18* was narrowed down to a 157-kb region on chromosome 3 that contains seven exonic non-synonymous SNPs. Six of these SNPs are located on two NBS-LRR genes, making them the best candidates of the causal gene in *RpsHC18*. Consequently, two SNP markers (Table 2) were developed for marker-assisted breeding for *P. sojae* resistance (Zhong et al. 2018a).

**Table 2:** Potential universal markers for marker-assisted breeding

Trait	Allele	Allele phenotype	Donor accessions^a^	Potential universal markers^b^	Details for markers	References
Biological nitrogen fixation						
Super-nodulation	*SS2*-*2*	2–3 times more nodules compared with normal soybean cultivars	SS2-2 (mutant of Sinpaldalkong 2)	A-specific allele Fw: CAACCTCACCGGCGTACTTCCGA Rv: CCTCAGCGTCTTCAACTTCGACAAACTC T-specific allele Fw: GAACAACCTCACCGGCGTACTTCCTT Rv: CCTCAGCGTCTTCAACTTCGACAAACTC	The ‘A-allele’ encodes a full-length GmNARK protein. The ‘T-allele’ encodes a truncated GmNARK protein which is the mutant allele from SS2-2	Kim et al. (2005)
Abiotic stress tolerance						
Salt tolerance	*GmCHX1*	Salt-tolerant	W05 (Qi et al. 2014), Tiefeng 8 (Guan et al. 2014)	Sequence marker: Conserved coding sequence of GmCHX1 Fw: ATGACGTTCAACGCGAGCACC Rv: TTAAAGTTCTTCGATAGCATCTT	Encodes the same protein sequence as GenBank accession KF879911, needs to confirm with Sanger sequencing	Qi et al. (2014)
Phosphorus efficiency	*GmHAD1*	Tolerance to low-phosphorus stress	HC 2	*qP10*-*2* Fw: CATTTATCATGTGAAAGCAG Rv: AACCAAAACAATCCAAGTAT		Cai et al. (2018d)
Low temperature	*qCTR*-*A2*	High seed yielding ability at low temperature	Toyoharuka	Sat_181, Sat_162, Sat_233	*qCTR*-*A2* is located near Sat_162	Ikeda et al. (2009)
Low temperature	*GmIRCHS*	Prevent low temperature induced seed coat discoloration	Toyoharuka	Toyoharuka specific Fw: GAGTTTGAAAAATGTATTCTTTCTCTTCC Rv: GTATCGCAGATTCCTCCTGC Toyomusume specific Fw: GCAAACCAAATCAAGTAAGAGCG Rv: CCCATTCCTTGATTGCCTTA	*GmIRCHS* is a candidate gene for the I allele at the *I* locus.	Ohnishi et al. (2011)
Biotic stress tolerance						
*Phytophthora sojae*	*RpsHC18*	*Phytophthora sojae resistance*	Huachun 18	BARCSOYSSR_03_0254 (0.3 cM) and BARCSOYSSR_03_0272 (0.7 cM)		Zhong et al. (2018a)
*Phytophthora* root rot	Glyma.02g245700 Glyma.02g245800 Glyma.02g246300	*Phytophthora* root rot resistance	Zaoshu18	ZCSSR33 Fw: TAGTTGATAGCACCTGGGGACA Rv: TTCTCAGTCTCAAATGCC ZCSSR46 Fw: AAAGGGAGAAGCAAGTAAT Rv: TCGCAAACAGTAAACACG		Zhong et al. (2018b)
*Phytophthora sojae*	*Rps1d*	PSR resistance	PI 103091	Satt152-Sat_186	*Rps1d* is located at 5.7 cM from Sat_186	Sugimoto et al. (2008)
Soybean cyst nematode	*rhg1*s*, rhg1g*	SCN resistance to race 1 and 3	PI84751(*rhg1*s), Gedenshirazu (*rhg1g*)	Satt309 and Sat_210	Both *rhg1*s and *Rhg4* are required for SCN resistance to race 1.	Kato et al. (2017), Suzuki et al. (2011)
Soybean cyst nematode	*Rhg4*	SCN resistance to race 1	PI84751	Satt632, Sat_162 and Sat_157	Both *rhg1*s and *Rhg4* are required for SCN resistance to race 1.	Kato et al. (2017), Suzuki et al. (2011)
Soybean mosaic virus	Glyma.13g194700 and Glyma.13g195100	Resistance to Soybean mosaic virus SC20	Qihuang-1	gm-ssr_13-14 Fw: ACAAAACGACAATTTTACCCA Rv: CGACCGGATACTGTAAAAGA gm-indel_13-12 Fw: GGTGAGTTTGGTCGATAATGT Rv: AGACTCGCGTAAGGTGTTCT	Expected amplicon size of gm-ssr_13-14: 181 bp Expected amplicon size of gm-indel_13-12: 484 bp	Karthikeyan et al. (2018)
Soybean mosaic virus	*Rsv3*	Resistance to Soybean mosaic virus strain C and D	Harosoy, Fukuibuki, Dewamusume, Suzuyutaka	BARCSOYSSR_14_1414, BARCSOYSSR_14_1416, BARCSOYSSR_14_1392, BARCSOYSSR_14_1403, M3SattM Fw: GATTGAAGGGTCACCAATCG, Rv: CGCTATCATCCAATGACCAA		Kato et al. (2016)
Peanut stunt virus	*Rpsv1*	Resistance to two PSV isolates, PSV-K and PSV-T	Fukuyutaka, Harosoy, Hyuga	Satt435-Sat_244		Saruta et al. (2011)
Common cutworm	*CCW*-*1*	Antibiosis resistance to common cutworm	Himeshirazu	Satt220–Satt175 CCW1_15 Fw: GGGAAACAGGGCAAGGAAC Rv: CAAGTGTTGTCTCGAATGGAAA		Komatsu et al. (2005)
Common cutworm	*CCW*-*2*	Antibiosis resistance to common cutworm	Himeshirazu	Satt567–Satt463		Komatsu et al. (2005)
Common cutworm	*qRslx1*	Antixenosis resistance to common cutworm	Himeshirazu	CCW1_15 Fw: GGGAAACAGGGCAAGGAAC Rv: CAAGTGTTGTCTCGAATGGAAA	QTL for antixenosis resistance, *qRslx1*, is close to *CCW*-*1.*	Oki et al. (2018)
Common cutworm	*qRslx2*	Antixenosis resistance to common cutworm	Himeshirazu	Sat_218		Oki et al. (2018)
Nutritional values						
Seed oil content	*qOIL_8_1*	Increased oil content	Jidou 12	Satt177 Satt341		Yan et al. (2019)
P34 allergen content	Low P34	Low P34 content	PI567476, PI603579A	Fw: CAAACTGTCATTCCCTGTTGG Rv: AAGAAGCAACACAAGGAAACC	Size of low P34-content allele larger, migrates more slowly on the gel	Jeong et al. (2013)
Morphological traits						
Seed Coat color	*I*	Non-pigmented seed coat	All accessions with non-pigmented seed coat	PCR marker: Inversion junction Fw: GGCCTGTTGTAGTGGAAAATTC Rv: CCTGGATACAGGTACGTTTTACT	An inversion in the *I* locus corresponds to the non-pigmented seed coat phenotype	Xie et al. (2019)
Plant height	*GmDW1*	Dwarf	Zp661	SNP08-1 Fw: TGCACCAAAACCAGCTCAAT Rv: AGGATCAGAAGGCTTGGGAC		Li et al. (2018b)
Growth Habit	*Dt1*	Indeterminate growth	Moshidou Gong 503	CAPS marker: TFL1b-pro Fw: CCATGCTTAATCGGCATCACT Rv: GGTGGTGGCATAGTTTAATT dCAPS marker: TFL1b-ex4 Fw: GGCTGCTGTCTACTTCAATGTCTAG Rv: GCCACATGTGAAGATCAACTTCCA	Digestion of TFL1b-pro amplification product of *Dt1* allele with *Nde*I gives 217 and 193-bp fragments Digestion of TLF1b-ex4 amplification product of *Dt1* allele with *Xba*I gives 155- and 21-bp fragments	Liu et al. (2010a)
	*dt1*	Determinate growth	Misuzudaizu			
Internode distance	*qSI13*-*1*	Short internode, lodging resistance	Y2	Sat_375–Satt657		Oki et al. (2018)
Lodging	*qLS19*-*1*	Lodging tolerance	Toyoharuka	Sat_099		Yamaguchi et al. (2014)
Seed Shattering	*pdh1*	Shattering resistance	Hayahikari, Harosoy, Wasekogane, Karlyutaka, Jack, Yukihomare	Dir_CAPS Fw: GCCCTCGTTGTGTTCTTCAT Rv: GCGTTGCTTCCGTTGTAGAT Dir_SNP_ARMS Outer forward: CTCTTGGCATAGCTAGGGAAAAGCACTA Outer reverse: GAAAACCACTATGTTTCCGAAGTGGAAC Inner forward: GAAGAGGCCACAACATGCACCATACT Inner reverse: TGTCGTGGAAGAAGAGGACTAAGTGTTT	SR: can be digested with *NheI* SS: cannot be digested with *NheI* Common band: 262 bp SR: 169 bp (SNP: T) SS: 146 bp (SNP: A) SS = shattering sensitive	Funatsuki et al. (2014)
Isoflavone content	*qIF5*-*1*	Isoflavone-related traits	Wayao	Bin799–800 on chromosome 05		Cai et al. (2018c)
Developmental stages						
Flowering time	*E11*	Flowering time and maturity	Minsoy (PI27890) and Archer (PI54687)			Wang et al. (2019)
Flowering time	*qFT12.1*	Delay flowering time	Wild soybean JWS156-1	BARCSOYSSR_12_0220 and BARCSOYSSR_12_0368 Satt192		Liu et al. (2018a)
Flowering time in tropical region	*j* (HT1 m)/ *j*-*4*	Delay flowering time in inductive short-day	Huaxia 3, etc	J‐Indel‐spanning ‐Fw GGTTTTGTGATGTGATCGGAGCC J‐Indel‐spanning‐Rv AGCCACATTTGTCTGGTGCTGATTTCC	A T>A indel results in a truncated *GmELF3* gene product	Yue et al. (2017)
Flowering time in tropical region	*j*	Delay flowering time in inductive short-day	PI 159925, BR121, etc	J‐620F (haplotype analysis) CTATTGTGTGAGTGAGATTGATTTGGAT J‐5190R (haplotype analysis) CTACTCTTTCGGGTAAAGCAATTACTAC	Detection of mutations on *GmELF3*	Lu et al. (2017), Yue et al. (2017)
Flowering time	*e1*-*fs, e1*-*as, e1*-*nl, e1*-*re*	Flowering time and maturity	Sakamoto wase (*e1*-*fs*), Harosoy and Williams 82 (*e1*-*as*), Toyosuzu and Toyomusume (*e1*-*nl*), Gokuwase-Kamishunbetsu (*e1*-*re*)	The *e1*-*as* and *e1*-*fs* are discriminated as shorter digested fragments from other alleles by the *Taqα*I and *Hinf*I digestion of PCR products generated with specific primers G33snpTaqcutF TCAGATGAAAGGGAGCAGTGTCAAAAGAAGT G33snpTaqcutR1 TCCGATCTCATCACCTTTCC The *e1*-*re* and *e1*-*nl* alleles can be distinguished by amplification of a shorter fragment and no amplified product from other alleles using mixture of three primers E1M0535_Fw CCGTTTGATTGGTTTTTGGT E1P0305_Rv CCCTTCAGTTTCTGCAGCTC e1re_0188Rv GAGAAGACAAACAATTCGAG		Tsubokura et al. (2014), Xia et al. (2012)
Flowering time	*e1*-*like*-*b* (*e1lb*)	Incandescent long daylength insensitivity	Zeika, Yubileinaya, Sonata	dCAPS Fw: GTGTAAACACTCAAAGTCCTT Rv: CGTCTTCTTGATCTTCCAACG PCR fragment of the loss-of-function *e1lb* allele is digested into two fragments by restriction enzyme of *Hpy*CH4IV	Glyma.04G143300 is considered as the candidate gene of *e1lb*	Zhu et al. (2019)
Flowering time	*e2*-*ns*	Flowering time and maturity	Enrei, Harosoy, Toyomusume, many others	SoyGI_dCAP_Dra_Fw GAAGCCCATCAGAGGCATGTCTTATT SoyGI_dCAP_Dra_Rv GAGGCAGAGCCAAAGCCTAT PCR fragment of *e2*-*ns* can be digested with *Dra*I.		Tsubokura et al. (2014), Watanabe et al. (2011)
Flowering time	*e3*-*Mo, e3*-*tr*	Flowering time and maturity	Moshidou Gong 503 (*e3*-*Mo*), Enrei (*e3*-*tr*)	The *e3*-*tr* with a smaller PCR fragment can be distinguished from the other alleles using primer mixture of E3_08557Fw: TGGAGGGTATTGGATGATGC E3_09908Rv: CTAAGTCCGCCTCTGGTTTCAG E3Ha_1000Rv: CGGTCAAGAGCCAACATGAG e3tr_0716Rv: GTCCTATACAATTCTTTACGACG Further, the *e3*-*Mo* allele was distinguished from others by the *Mse*I digestion of the PCR products using the primers E3_08094Fw: TTGCATGAAGTTTTGGTTGC E3_08417Rv: CAACTGAACTGAAGACCCACAA		Tsubokura et al. (2014), Watanabe et al. (2009)
Flowering time	*e4*-*SORE*-*1*	Flowering time and maturity	Hayahikari, Yukihomare, Kariyutaka	The *e4*-*SORE*-*1* allele can be distinguished from *E4* by allele-specific primer pairs PhyA2-Fw: AGACGTAGTGCTAGGGCTAT PhyA2-Rv/E4: GCATCTCGCATCACCAGATCA PhyA2-Rv/e4: GCTCATCCCTTCGAATTCAG		Liu et al. (2008), Tsubokura et al. (2014)
Flowering and maturity	*e9*	Late flowering time and maturity	TK780, Shensei	Interval between Satt215 and Satt431 M5-indel-30600002- Fw: TGAAGCATTGTCCCCTGTTTCTA Rv: GGTATTTGTCATCATGGCATCCA M7-indel-30845759 Fw: AGGATGAAAAGAGAAGATGTTG Rv: AGATTTTCTTGATAGAATAGTAACG.	*GmFT2a* (Glyma16 g26660) or *GmFT2b* (Glyma16g26690) are considered as the candidate genes of *E9*	Kong et al. (2014)
Flowering time	*qDTF*-*J*	Early flowering	Sakamoto wase, Otome wase	Interval between SSR-J5 and SSR-FT3a SSR-J5 Fw: CAATATGACTGGAGGCTCATGA Rv: CCTAAGTAGGCCTACCAAT SSR-FT3a Fw: AAGCAGTGTGACTCAGTGAA Rv: GGACATCAGGATCCACCAT	*GmFT5a* (Glyma.16G044100) is considered as the candidate gene	Takeshima et al. (2016)
Maturity	*qdfm1*	Short reproductive period	Ippon-Sangoh	Interval between Sat_128 and Satt583, Satt519 is closest		Komatsu et al. (2011)
Maturity	*qdffgm1*	Short reproductive period	Peking	Interval between SSR markers s008000014-2 and T001111280 m, s008000014-2 is closest. s008000014-2 Fw: TACAACTTTGATGTTCCCATCTT Rv: AAGCTCCTTCCTCTCAAGTTGTT T001111280 m Fw: GGTGCTCTTCCTCACATTAGAGA Rv: TAGGTTGCAAGTATAGCGTGTTT		Watanabe et al. (2018)

^a^Only major cultivars used in the reference study will be listed

^b^Fw, forward primer; Rv, reverse primer; primer sequences are read from 5’ to 3’

China has its own system of classifying soybean mosaic virus (SMV), which designates the 22 SMV strains in China as SC1–SC22. Among them, SC20 is the most widespread in the five provinces in southern China, affecting soybean cultivation in that region. A SMV resistance QTL was fine-mapped to a 79-kb region on chromosome 13 of Qihuang-1, flanked by a new SSR marker and a new INDEL marker (Table 2) (Karthikeyan et al. 2018). Two TIR-NB-LRR protein-encoding genes that are highly polymorphic between the two parents in the mapping population are identified within this QTL, and they are highly likely to be the candidates of the SC20 resistance gene (Karthikeyan et al. 2018).

To introduce the resistance against *Micosphaera diffusa* Cooke & Peck, which causes powdery mildew disease (PMD), to Chinese soybean cultivars, researchers have made use of the PMD-resistant soybean cultivar BRSMG68 (B13) from Brazil (Jiang et al. 2018). The PMD resistance locus was mapped to a 188-kb region on chromosome 16 of B13 that contains 17 disease resistance genes accounting for 78% of the phenotypic differences within the mapping population (Jiang et al. 2018).

Alterations in plant architecture have played an important part during the Green Revolution to improve grain yield. Through EMS mutagenesis, a dwarf mutant (*dw*) was found in the Chinese soybean cultivar Zhongpin 661 background (Li et al. 2018b). The causal mutations were found on a gene encoding the ent-kaurene synthase, which disrupted the biosynthesis of gibberellins (Li et al. 2018b). Although the dwarf allele showed its potential for breeding new cultivars with high harvest index (Table 2), the actual effect of the mutation on yield has not yet been fully assessed.

Soybean cultivation has expanded beyond its natural habitat since domestication. Since soybean production is highly sensitive to photoperiod, efforts have been spent on studying the flowering and maturation time of soybean to maintain the yield at different latitudes. *E1*–*E4* are the well-characterized major loci controlling the flowering and maturation times of soybean (Jiang et al. 2014; Miladinovic et al. 2018; Tsubokura et al. 2014). In a recent study, using two genetic populations with fixed genotypes in the *E1*–*E4* loci, researchers were able to identify two other loci in soybean contributing to flowering and maturation, specifically in chromosome 4 of the Dongnong 50 × Wm82 RIL population and in chromosome 6 of the Suinong 14 × Enrei RIL population, respectively (Kong et al. 2018). However, these two QTLs are possibly linked with the other *E* loci, making the dissection of them difficult.

When grown in the tropical region, temperate soybean cultivars tend to flower before accumulating a reasonable amount of biomass, thus reducing the yield. Therefore, those soybean cultivars possessing the recessive long-juvenile (LJ) trait which allows them to stay vegetative for longer under inductive short-day conditions would be desirable for that region. The *J* locus controlling the long-juvenile trait was mapped to chromosome 4 on the soybean genome (Table 2) (Lu et al. 2017; Yue et al. 2017). Recessive *j* alleles were found to be actually *GmELF3* carrying either frameshift or missense mutations that either abolish or diminish the functionality of the encoded protein (Lu et al. 2017; Yue et al. 2017). Manipulation of FT homologs in soybean provides another way to generate germplasm with longer growth period and higher yield in tropical region. Liu et al. (2018b) overexpressed *GmFT1a*, a flowering inhibitory gene, and created materials with delayed flowering and maturity and increased biomass and yield in short-day condition (Liu et al. 2018b). Cai et al. (2019) employed the CRISPR/Cas9 system to specifically knock out the soybean flowering-promoting genes *GmFT2a* and *GmFT5a* and the *ft2a ft5a* double mutant flowered about 31 days later than that wild type and produced more pods and seeds (Cai et al. 2019). Three Chinese soybean cultivars Longhuang #1-3 were developed by screening for the presence of the functinonal *GmCHX1* gene (Qi et al. 2014) in drought tolerant accessions (Table 2), resulting in highly adaptive soybeans for improving agricultural productivity on marginal lands.

Pod distribution on the soybean plant also affects the harvest index and is influenced by a number of minor-effect loci. Using 7-year worth of data, a study identified 11 major QTLs and 90 epistatic pairs controlling the first pod height (Jiang et al. 2018). Another study identified 47 QTLs associated with the distribution of pods with different numbers of seeds at the upper, middle, and lower sections of the soybean plant (Ning et al. 2018). It appears that pod distribution is governed by a large number of QTLs that are difficult to be introduced using simple marker-assisted selection. Instead, genomic selection would be a better way of selecting supreme offsprings with a desirable pod distribution pattern.

Two Chinese cultivars with contrasting oil and protein contents, Linhefenqingdou and Meng 8206, were used to build an RIL population for QTL mapping (Karikari et al. 2019). With data from six different environmental conditions, 44 main-effect QTLs for protein and oil contents were identified in an environment-dependent manner (Karikari et al. 2019). Fifteen of the QTLs were novel, while 20 had *R*^2^ > 10%. Among them, *qPro*-*7*-*1* was a new QTL on chromosome 7 that was consistently detected in three individual environmental conditions and in the combined environment, accounting for at least 13.6% of the total variations (Karikari et al. 2019). *qOil*-*8*-*3*, *qOil*-*10*-*2*, and *qOil*-*10*-*4* were detected in at least two individual environmental conditions and in the combined environment, explaining 6.2–30.6% of the total variations (Karikari et al. 2019). There were significant additive interactions among seven of these loci, as well as interactions between themselves and the environmental conditions, while epistasis was observed in three loci pairs (Karikari et al. 2019).

Phosphorus (P) is a limiting macronutrient that the soybean can only obtain from soil deposition and fertilizer. Therefore, increasing P-efficiency in soybean would improve the adaptability of soybean to low-P environments. QTL mapping for the P-efficiency locus was done using an RI population originated from HC 2 and Wayao (Cai et al. 2018d). HC 2 was bred from the Chinese cultivar Guizao1 and the Brazilian cultivar CONFIANGA with high P-efficiency, while Wayao is a Chinese landrace with low P-efficiency. Fifteen major QTLs were identified, with *qP10*-*2* at chromosome 10 explaining the highest percentage of variations (13.98%) (Cai et al. 2018d). This QTL contains an acid phosphatase-encoding gene, *GmHAD1*. Ectopic expression and overexpression of *GmHAD1* in Arabidopsis and soybean hairy root were both shown to improve P-efficiency (Cai et al. 2018d).

To enhance the isoflavone contents in soybean seeds, a QTL mapping study identified 108 loci in a biparental population (Cai et al. 2018c). Fifteen of these QTLs were consistently found in different environmental conditions, with each explaining at least 1.8% of the variations (Cai et al. 2018c). Among them, *qIF5*-*1*, spanning a 611-kb region on chromosome 5, could explain 6.37–59.95% of the observed variations in the acetyldaidzin, daidzin, genistin, daidzein, glycitin, malonyldaidzin, malonylglycitin, malonylgenistin, genistein, and total isoflavone contents in the mapping population (Cai et al. 2018c). Although the causal gene of this QTL is still unknown, this QTL could be introduced into cultivars to improve isoflavone contents using MAS.

### Progress on soybean molecular breeding in Japan

In Japan, soybeans are now cultivated in paddy fields in place of rice to prevent the overproduction of rice, according to a government policy. As a result, many problems related to lowland conditions have caused widely fluctuating and low yields in Japanese soybean production. Resistance genes to the widespread disease caused by *Phytophthora sojae* and *Calonectria crotalaria*, as well as seed-flooding tolerance at the germinating stage, have become very important for achieving higher yields. In addition, due to the widespread mechanization of farm operations, improvement in plant adaptation to high plant density and in the resistance against pod shattering during machine harvesting is desired goals. To this end, four cultivars with pod dehiscence resistance, ‘Sachiyutaka A1 gou’ (Hajika et al. 2016), ‘Fukuyutaka A1 gou’ (Yamada et al. 2017), ‘Enreinosora’ (Yamada et al. 2017), and ‘Kotoyutaka A1 gou’ (MAFF, Japan), have been developed by MAS through repeated backcrossing of progenies with the pod-shattering-resistant *pdh1* mutant originating from ‘Hayahikari.’ Introduction of *pdh1* into other leading Japanese soybean cultivars by MAS is underway.

The soybean cyst nematode (SCN, *Heterodera glycines* Ichinohe) is one of the most damaging pests of soybean. SCN races 1, 3, and 5 have been reported in Japan. Soybean mosaic virus (SMV), soybean dwarf virus (SbDV), southern bean mosaic virus (SBMV), and peanut stunt virus (PSV) are also considered to be serious pathogens affecting seed quality. In southwestern Japan, significant damage to soybean plants is caused by the common cutworm (CCW, *Spodoptera litura* Fabricius). Therefore, breeding resistance into the crop against these pests and pathogens is essential for improving soybean yield.

There are two main SCN resistance resources for breeding in Japan. The Japanese cultivar ‘Gedenshirazu’ (PI 561360) has the SCN resistance QTLs *Rhg4/rhg1g/rhg2g*, while the Korean cultivar LG-59 (PI84751) has *Rhg4/rhg1*s*/rhg2*s. *Rhg4*, *rhg1*s, and *rhg2g* or *rhg2*s are necessary for race-1 resistance, and either *rhg1*s or *rhg1g* is necessary for race-3 resistance. *rhg1g* of ‘Gedenshirazu’ and *rhg1*s of PI84751 are allelic on chromosome 18, while *rhg2g* of ‘Gedenshirazu’ and *rhg2*s of PI84751 are non-allelic but are located close to each other on the same chromosome (Suzuki et al. 2011). SMV in Japan is classified into five major strains, A, B, C, D, and E (Takahashi et al. 1980). Many Japanese varieties are resistant to the A and B strains on account of existence of *Rsv1*, while resistance to the C and D strains has been limited. *Rsv3* derived from ‘Harosoy’ on chromosome 14 has been confirmed to confer resistance to the Japanese SMV strains C and D by MAS using markers linked to *Rsv3* (Kato et al. 2016). *Rps1d* and *Rps1k* for *Phytophthora sojae* resistance (PSR) are the most effective resistance genes among 14 *Rps* genes in Japan by using 109 *P. sojae* isolates from 14 regions (Moriwaki 2010). Sugimoto et al. (2008) mapped *Rps1d* to chromosome 3 using progenies from a cross between ‘Tanbakuro’ and ‘PI 103091’ to determine selectable SSR markers (Sugimoto et al. 2008). PSV resistance is traced to a single dominant gene, *Rpsv1*, near *Satt435* on chromosome 7 by using RILs with PSV resistance from a cross between ‘Hyuga’ and ‘Enrei’ (Saruta et al. 2011). Two QTLs for antibiosis resistance to CCW, *CCW*-*1*, and *CCW*-*2*, both on chromosome 7, have been identified using progenies from a cross between ‘Fukuyutaka’ and ‘Himeshirazu’ (Komatsu et al. 2005). Two QTLs, *qRslx1* and *qRslx2*, controlling antixenosis resistance have been identified from the same population (Oki et al. 2011).

By integrating the above information, the resistance genes against SCN, SMV, and SbDV can be incorporated into one target cultivar by high-resolution PCR fragment analyses using a florescent sequence machine (Kato et al. 2017). There has been a certain degree of success in this endeavor. For example, ‘Yukihomare R’ with resistance to SCN races 1 and 3 was developed by MAS for *rhg1*s and *Rhg4* from PI 84751 and repeated backcrossing with ‘Yukihomare’, a leading cultivar in Hokkaido (Suzuki et al. 2017). In contrast, ‘Suzumaru R’ with resistance to SCN races 1 and 3 was developed by MAS for *Rhg4* and *rhg1*s derived from PI 84751 and *rhg2g* from ‘Gendenshirazu’ through repeated backcrossing with ‘Suzumaru’, a leading cultivar for natto in Hokkaido (Kurosaki et al. 2017). ‘Fukuminori’ is developed by the introgression of two QTLs for antibiosis resistance, *CCW*-*1* and *CCW*-*2*, into ‘Fukuyutaka’ by MAS with repeated backcrossing (Oki et al. 2011). Nearly all the characteristics of ‘Fukuminori’ are the same as those of ‘Fukuyutaka’ except for a slightly smaller seed size and the resistance to CCW. The SMV-resistant ‘Hyoukeikuro4 gou’ (MAFF, Japan) and PSR-resistant ‘Hyoukeikuro5 gou’ (MAFF, Japan) were developed by MAS and repeatedly backcrossed with a large black-seeded cultivar ‘Hyoukeikuro3.’

The combination of alleles at maturity-related loci is very important to develop a new cultivar with early harvesting for rotations with different crops or to adjust maturity of a new variety which can adapt to environments at different latitudes retaining the same seed quality with the similar genetic background. Among ten major classical genes, *E1*–*E9* and *J*, *E1* (Xia et al. 2012)*, E2* (Watanabe et al. 2011)*, E3* (Watanabe et al. 2009)*, E4* (Liu et al. 2008), *E9* (Kong et al. 2014), and *J* (Lu et al. 2017) have been isolated. In addition, responsible genes for *E1*-*like*-*b* (Zhu et al. 2019) and *qDTF*-*J* (Takeshima et al. 2016) have been reported. Two QTLs, *qDfm1* (Komatsu et al. 2011) and *qDFFGm11* (Watanabe et al. 2018), have been identified as QTL controlling post-flowering period on chromosome 11. Allelic variation of *E1, E2, E3,* and *E4* genes in the major soybean germplasm has been examined by using allele-specific DNA markers (Tsubokura et al. 2014).

Chilling tolerance in northern Japan, drought and high-temperature tolerance in southwestern Japan, and waterlogging tolerance throughout the whole country are also important for Japanese soybean. The cold (CD) tolerance QTL was mapped to chromosome 8 near the *GmIRCHS* gene by using two sets of RILs between a CD-tolerant ‘Toyoharuka’ and CD-susceptible cultivars (Ohnishi et al. 2011). The markers developed are currently used in MAS in Hokkaido. The QTL for the high seed yield trait at low temperature of ‘Toyoharuka’ was mapped near *Sat_162* on chromosome 8 by using the RILs of ‘Toyoharuka’ × ‘Toyomusume’ (Ikeda et al. 2009). By using these same RILs, a research group found that the lodging tolerance of ‘Toyoharuka’ is controlled by a major QTL, *qLS19*-*1*, that is not associated with maturity or growth habit on chromosome 19 (Yamaguchi et al. 2014). Another QTL on chromosome 13, *qSI13*-*1*, the determinant of short internodal length and reduced plant height of the cultivar, ‘Y2’ (Oki et al. 2018), is being used to control plant height to prevent lodging.

### Progress on soybean molecular breeding in the Republic of Korea

Officially, soybean breeding started in Korea with the release of the first landrace, Jangdanbaekmok in 1913. During the past 100 years, more than 178 soybean cultivars have been developed through hybridization-based breeding (87%) and registered with the two Korean national institutes, the RDA-Genebank Information Center (http://www.genebank.go.kr) and the Korea Seed and Variety Service (http://www.seed.go.kr). Most of the cultivars have been registered during the last 30 years, suggesting that the availability of genetic and genomic information has accelerated the soybean breeding efforts in Korea (Kim et al. 2011). Efforts of soybean breeding in the Republic of Korea have been dedicated to the improvement in nutritional values, disease and stress resistance, yield, and nitrogen fixation.

Soybean yield usually consists of three major components: the number of pods per plant, the number of seeds per pod, and seed weight. It has been found that the more branches the plant has, the higher will be the number of pods and thus the yield. One out of five branch number-associated quantitative trait nucleotides (QTNs) in the Korean soybean collection was found to overlap with a major branch number QTL, *qBR6*-*1*, which contains the gene *BRANCHED1* (*BRC1*; *Glyma.06g210600*). Specifically, one missense mutation and two SNPs upstream of *BRC1* were linked to branch numbers in 59 soybean accessions. *BRC1* encodes the transcription factors TEOSINTE-BRANCHED1/CYCLOIDEA/PROLIFERATING CELL FACTORS (TCP) and functions as a regulatory repressor of branching (Shim et al. 2019). Additionally, it has been proposed that the number of seeds per pod is directly linked to narrow leaflets in soybean. The mapping of the BC3F2 population from a cross between two cultivars, Sowon (narrow leaflets and high number of seeds per pod) and V94-5152 (broad leaflets and low number of seeds per pod), showed that Sowon carries a recessive allele of the *ln* gene on chromosome 20 (Jeong et al. 2012). The transition from broad (Ln) to narrow leaflet (ln) is associated with an amino acid substitution in the EAR motif encoded by a gene designated as *Gm*-*JAGGED1* (*Glyma20g25000*), which is homologous to the Arabidopsis *JAGGED* (*JAG*) that regulates lateral organ development and its variant exerts a pleiotropic effect on fruit patterning (Jeong et al. 2012). Later studies by the same group suggested that *Glyma.08g281900* could be the Lf1-encoding gene that controls lateral organ development. It was concluded that this gene possibly exerts a pleiotropic effect on the number of seeds per pod (Jeong et al. 2017), making it a good target for breeding.

While the soybean seed is nutritious, it also contains several anti-nutritional factors that are harmful to both humans and livestock. For example, the seed protein, P34, is a potential allergen which causes complications in soybean-sensitive patients. To develop lines with low P34, PI567476 was crossed with the Korean cultivar, Hwanggeum, and the resulting F2 and F3 populations were studied for the identification of molecular markers for the three copies of the gene encoding P34, i.e., *Glyma08g12270*, *Glyma08g12280*, and *Glyma05g29130*. As a result, a molecular marker (Table 2) and a polyclonal antibody were developed successfully for the selection of low P34-expressing lines (Jeong et al. 2013).

The beany flavor of the soybean seed is due to the seed lipoxygenase isozymes, LOX1-3, with LOX2 being the biggest contributor to the beany flavor, which is sometimes undesirable. Jinpumkong 2, a Korean soybean cultivar, lacks all three lipoxygenases (having the null alleles, *lx1*, *lx2*, and *lx3*). Using the RILs of a cross between Pureunkong 9 and Jinpumkong 2, a 175-bp fragment was identified within the *Lx2* gene on chromosome 13 that is retained in *G. soja* but has become rare in the *G. max*. A single-nucleotide-amplified polymorphism (SNAP) marker was subsequently made available for soybean breeders to facilitate the identification of cultivars/lines lacking LOX2 (Shin et al. 2008, 2012).

To improve the oxidative stability and quality of soybean oil, breeding programs have mainly focused on reducing the saturated fatty acid and linolenic acid contents and increasing oleic acid in the oil. Hence, delta-12 fatty acid desaturase 2 (FAD2), which converts oleic acid (18:1) to linolenic acid (18:2), becomes the target for modification via molecular breeding. There are two *FAD2* loci, *FAD2*-*1A* (*Glyma10g42470*) and *FAD2*-*1B* (*Glyma20g24530*), in the soybean genome. To develop high-oleic-acid lines (> 80% of total oil content vs. an average of 20-50% among existing soybean cultivars), efforts were made to combine a recessive mutant allele at the *FAD2*-*1A* locus (*Glyma10g42470*) and a recessive mutant allele at the *FAD2*-*1B* locus (*Glyma20g24530*) using molecular marker assays (Pham et al. 2011). In a later study, a non-synonymous SNP on *FAD2*-*1A* (S117 N) from 17D, an EMS mutant of Wm82, and another one on *FAD2*-*1B* (P137R), a mutant allele from PI 283327, were identified to be highly associated with the high-oleic-acid phenotype (Kulkarni et al. 2018). Similarly, an EMS mutant, PE1690, derived from the cultivar, Pungsannamul, was found to be low in linolenic acid. The phenotype was the result of a single-base mutation (W128*) in the *GmFAD3A* gene on the locus *Glyma14g37350*, which rendered the desaturase enzyme non-functional (Kim et al. 2015). A derived cleaved amplified polymorphic sequence (dCAPs) assay specific for this artificial polymorphism was thus developed for future breeding activities (Kim et al. 2015).

Soybean contains significant amounts of bioactive secondary metabolites, particularly soyasaponins. Studies in Korea reported natural variations in saponin contents in wild soybeans and identified different group A acetylsaponin-deficient mutants (Krishnamurthy et al. 2014a, b, c; Panneerselvam et al. 2013). Apart from saponins, other compounds such as soy isoflavones, polyphenols, flavonoids, proanthocyanidins, and phenolic acids have also been analyzed in large-scale wild soybean collections in Korea as well as in wild soybeans from other natural habitats. These studies identified unique germplasm accessions with higher amounts of these beneficial/desirable secondary metabolites that will be an important resource for future breeding (Ha et al. 2018; Nawaz et al. 2018; Tsukamoto et al. 2018). In additional to beneficial metabolites, trace harmful metabolites such as raffinose are also found in soybean. A novel allele of the putative soybean raffinose synthase gene, *RS2*, was discovered in PI200508, that was associated with low raffinose and stachyose contents. New Korean soybean cultivars, Daewon, Cheongja, and Danmiput, containing low levels of raffinose and stachyose were subsequently developed based on a specific marker assay for the PI200508 *RS2* allele that allows for the direct selection of the low-raffinose and stachyose phenotype (Yang et al. 2014).

As mentioned before, the genetic control of flowering time is very important in photoperiod-sensitive soybean. Both wild (IT182932) and cultivated (Wm82) soybean genomes were blasted against Arabidopsis genes related to photoperiod-dependent flowering time and 118 genes were singled out based on functional DNA variations and insights into duplicated regions (Kim et al. 2012). Using two RIL populations, Jinpumkong 2 × SS2-2 (J × S) and Iksannamulkong × SS2-2 (I × S), QTLs related to days to flowering (DF) (Liu et al. 2010b) and days to maturity (Liu et al. 2011) were identified. In total, 18 QTLs for DF and days to maturity along with six yield-related traits were isolated from these two RIL populations (Liu et al. 2011). Whole-genome resequencing has also been used to identify genes governing DF in an early-flowering soybean cultivar, Josaengserori (mutant from Seoritae). Among 30 DF-related genes detected using SNPs, *Glyma02g33040*, *Glyma06g22650*, *Glyma10g36600*, *Glyma13g01290*, *Glyma14g10530*, *Glyma16g01980*, *Glyma17g11040*, *Glyma18g53690*, and *Glyma20g29300* contained non-synonymous substitutions between Josaengserori and Seoritae. Among these genes, it was discovered that the changes in *Glyma10g36600* (*GI*), *Glya02g33040* (*AGL18*), *Glyma17g11040* (*TOC1*), and *Glyma14g10530* (*ELF3*) in Josaengserori affected the expression of *GmFT2a* and resulted in early flowering (Lee et al. 2016).

Since legume root nodules contain symbiotic nitrogen-fixing rhizobia, being able to increase nodule number could potentially increase biological nitrogen fixation. The cultivar SS2-2, having increased number of nodules, is an EMS mutant of a Korean soybean cultivar Sinpaldalkong 2. The causal mutation was found to be a nonsense mutation of *GmNARK*. Using this discovery, a PCR-based SNAP marker was developed for the identification of supernodulating soybean plants (Kim et al. 2005, Table 2). Genetic mapping has helped identify two nodule-specific genes, *GmPGN* (a polygalacturonase-encoding gene) and *GmEKN* (a short nodule-specific gene). The former was located near a known QTL conferring resistance to soybean cyst nematode (SCN) on the molecular linkage group (MLG) B1 and the latter on MLG A2 (Jeong et al. 2006). Studies on nodule development in other countries reported that three gene loci, *rj1*, *rj5*, and *rj6*, control the initial nodule development in soybean. Fine genetic and physical mapping of the non-nodulating locus, *rj1*, suggested that this simple recessive allele was created by a single-base-pair deletion from a spontaneous mutation, resulting in a premature stop codon and a non-functional NFR1α (nod factor receptor) (Lee et al. 2011b).

Cloning of a cluster of NBS-LRR resistance gene candidates from MLG F of the virus-resistant soybean line, PI96983, showed that this cluster contains multiple genes which interact to produce customized responses to different SMV strains. In order to improve SMV resistance in soybean cultivars, efforts have been made to identify potential resistance genes. Three independent *R* gene loci, *Rsv1*, *Rsv3*, and *Rsv4*, have been reported in different soybean cultivars to confer resistance to SMV strains G1–G7 (Kim et al. 2016) and are associated with a cluster of genes encoding NB-LRR proteins. Specifically, one candidate gene (*Glyma14g38533*) has been singled out in the *Rsv3* cluster as a possible causal gene (Redekar et al. 2016). These three SMV resistance loci confer variable levels of resistance in soybean. However, *Rsv4* in particular provides novel resources for map-based cloning and genetic improvement in soybean against SMV because it confers durable type resistance. The BC3F2 population (309 individuals from a cross between Sowon and V94-5152) were used to identify four SNPs perfectly associated with SMV resistance. Interestingly, haplotype analysis suggested that the *Rsv4* locus in *G. max* was recently introgressed from wild soybean. Therefore, if we want to find variations in *Rsv4* or the other two *Rsv* loci in order to fully understand the resistance mechanism of soybean against SMV, we must look to the wild soybean germplasms (Ilut et al. 2016).

Progress has also been made in the discovery of genes/loci associated with the resistance against other pests and pathogens. A novel QTL (*Raso 2*) for the Korean biotype foxglove aphid (*Aulacorthum solani*) resistance in soybean has been identified in the wild Japanese accession, PI366121, using GoldenGate SNP microarray (Lee et al. 2015). Phomopsis seed decay (PSD) caused by *Phomosis longicolla* results in major damage to seed quality and leads to yield loss. Two QTLs for resistance to PSD have been identified in an RIL population derived from a cross between the PSD-resistant cultivar, Taekwangkong, and the PSD-susceptible line SS2-2, namely *PSD*-*6*-*1* and *PSD*-*10*-*2* at the intervals *Satt100*–*Satt460* and *Sat_038*-*Satt243* on chromosomes 6 and 10, respectively. These newly identified QTLs will help improve soybean resistance to *P. longicolla* (Sun et al. 2013).

## Perspectives

According to the statistics published by the Food and Agriculture Organization of the United Nations, the per-hectare yield of soybean in the USA is 1.5–2 times those in Asian countries. This suggests that there is still room for the yields of Asian soybeans to improve, despite reaching a plateau in recent years. In addition to achieving higher yields, agriculture also faces challenges due to climate change. To produce high-quality soybean using methods that are acceptable to the consumer, traditional and advanced breeding techniques making use of mined genetic resources from germplasm collections are essential. The distribution and maintenance of these resources usually involve high costs and are influenced by national policies. China, Japan, and Korea, as the origins of soybean domestication, are the guardians of an appreciable amount of wild soybean resources. It would be of tremendous benefits to the rest of the world for these governments to facilitate the sharing of the materials across national boundaries for sustained soybean crop improvement.

Genome-wide association analysis, genomic selection, and genome editing will become essential tools for assessing favorable alleles among germplasms and combining them to develop new cultivars. Nowadays, high-throughput genome sequencing is no longer the limiting factor for population genomics. In addition, high-throughput phenotyping tools such as unmanned aerial vehicles with multiple sensors and advanced digital image processing techniques are being used to evaluate traits which previously could only be estimated using labor-intensive and destructive methods. Large-scale evaluations of such traits as spatial and temporal changes in canopy biomass, branching, lodging, light capture, and photosynthesis, which are highly correlated with productivity, are expected to provide new insights to guide molecular breeding.

Nevertheless, specific potentially adaptive alleles and genes may not already exist in nature even with the availability of large germplasm collections. New genetic resources could be created *de novo* by chemical/radiation mutagenesis and genome editing. The drawback for random mutagenesis by chemical or radiation is that the mutated alleles may cause pleiotropic effects and require detailed characterization, and the residual background mutations in the mutants will take time to be completely removed. However, these mutants can be useful as the foundation for genome editing, along with alleles from wild accessions. Nonetheless, current protocols for genome editing still rely largely on modifying existing elements in the genome rather than the creation of completely new genes.

## Author contribution statement

H-ML coordinated the writing. GZ, TH, GC, HN, and H-ML conceptualized the review. BJ, AK, and GC wrote the sections specifically related to China, Japan, and Korea, respectively. M-WL, ZW, and F-LW integrated the information and wrote the first draft. M-WL and H-ML wrote the final version.
